# Role of Host Genetic Factors in the Outcome of Hepatitis C Virus Infection

**DOI:** 10.3390/v1020104

**Published:** 2009-08-05

**Authors:** Bertram Bengsch, Robert Thimme, Hubert E. Blum

**Affiliations:** Department of Medicine II, University Hospital Freiburg, 79106 Freiburg, Germany; E-Mails: Bertram.Bengsch@uniklinik-freiburg.de (B.B.), Hubert.Blum@uniklinik-freiburg.de (H.B.)

**Keywords:** Hepatitis C, genes, natural history, HLA, T cells, NK cells

## Abstract

The natural history of hepatitis C virus (HCV) infection is determined by a complex interplay between host genetic, immunological and viral factors. This review highlights genes involved in innate and adaptive immune responses associated with different outcomes of HCV infection. For example, an association of HCV clearance with certain HLA alleles has been demonstrated. The mechanisms responsible for these associations have been linked to specific T cell responses for some particular alleles (e.g., HLA-B27). Genetic associations involved in T cell regulation and function further underline the role of the adaptive immune response in the natural history of HCV infection. In addition, some genes involved in innate NK cell responses demonstrate the complex interplay between components of the immune system necessary for a successful host response to HCV infection.

## 1. Introduction

**Figure 1.**
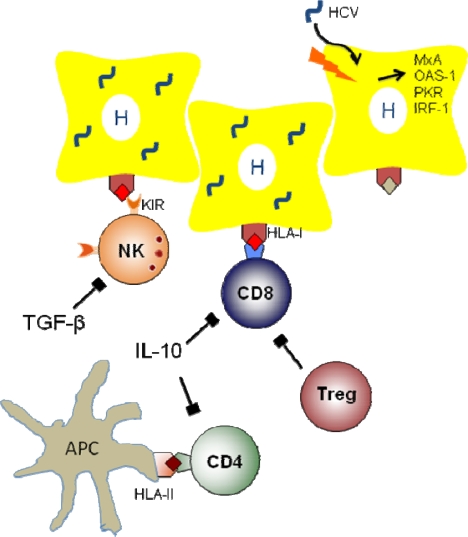
Schematic illustration of the components involved in the immune system against HCV based on evidence from genetic association studies.

Hepatitis C virus (HCV) is an RNA flavivirus currently infecting approx. 170 million people worldwide [1]. Acute HCV infection is asymptomatic in the majority of patients, but persists in about 70% of them. These patients with persistent liver inflammation are at risk of disease progression to liver fibrosis, liver cirrhosis and hepatocellular carcinoma (HCC) with potentially fatal outcomes. Currently, there is no effective vaccine against HCV available and antiviral treatment options have limited efficacy, especially in patients infected with HCV genotype 1 or 4, and have potentially severe side-effects. Thus, the understanding of the mechanisms that determine the natural course of HCV infection is of pivotal importance. In this review, the host genetic factors influencing the outcome (viral clearance *vs.* viral persistence) of HCV infection will be discussed. It is interesting to note that the genetic associations that have been identified in different cohorts worldwide pertain to genes that are prominently involved in the host antiviral immune response (depicted in Figure 1).

The host immune response to viral infections is characterized by various independent components. Next to physical barriers, innate immunity comprises soluble components (e.g., complement factors, type I interferons) and cellular components (e.g., granulocytes, macrophages, dendritic cells, natural killer (NK) cells). Adaptive immunity includes humoral components (antibodies produced by B cells) and, especially important in viral infections, cellular immune responses (CD4+ and CD8+ T cells).

Studies in humans and animal models of HCV infection have demonstrated that HCV elicits innate immune responses early after infection. However, the virus can persist in the face of the innate immune response. Indeed, viral clearance occurs only in the presence of antiviral CD4+ and CD8+ T cell responses [1, 2]. A successful T cell response requires the presentation of viral peptides bound to HLA molecules on the surface of antigen-presenting cells to T cells bearing a reactive T cell receptor (TCR). Importantly, HLA alleles are extremely variable in the human population and several HLA types have been identified that are associated with different outcomes of HCV infection, the most prominent one being the protective HLA-B27 allele.

In chronic HCV infection, the antiviral T cell response is typically dysfunctional. This is probably due to multiple coexisting mechanisms (reviewed in [3]) that include the presence and activity of regulatory T cells and an immunosuppressive cytokine milieu (IL-10). Studies that report associations between different polymorphisms in the IL-10 gene and IL-10 promoter regions support a role of the genetic background in modulating the function of regulatory cell subsets in HCV infection. In addition, alterations in T cell differentiation and function that may contribute to viral persistence have been demonstrated in chronic HCV infection. Of note, several genes involved in T cell differentiation have also been linked to different outcomes of HCV infection.

Here we will give an overview about the genetic factors and their impact on the natural course of HCV infection. The association of several genes with treatment response has been reviewed elsewhere [4] and will not be discussed here. It is important to bear in mind that most genetic association studies have been performed in different cohorts using different methodological approaches. Also, studies with relatively small patient cohorts may be unable to determine the role of single polymorphisms in the outcome of HCV infection due to the lack of statistical power. In addition, only few genetic studies addressing the natural history of HCV infection have been performed in which the sequence of the initial viral inoculum was known. Hence, viral factors that affect the outcome often cannot be excluded. Genetic association studies that are not supported by experimental evidence have thus to be interpreted with caution.

## 2. Review

### 2.1. Genes involved in innate immunity

Virus infection can elicit an immediate antiviral response in the infected cell, including the activation of the interferon (IFN) system [5]. IFNs induce several genes that encode for proteins with antiviral activity. Interestingly, Knapp *et al.* identified polymorphisms in IFN-induced genes, such as myxovirus resistance-1 (MxA), 2-5-oligoadenylate synthetase 1 (OAS-1) and double-stranded RNA-dependent protein kinase (PKR), which are associated with self-limiting infection [6]. While MxA polymorphisms were not associated with the outcome of HCV infection in another study [7], single nucleotide polymorphisms in the promoter region of the IFN regulatory factor-1 (IRF-1) were associated with protection from viral persistence [7]. These results indicate that differences in genes involved in early innate immunity responses may affect the natural course of HCV infection.

Currently, limited information is available regarding the role of host genetic factors involved in cellular innate immune responses by dendritic cells or macrophages. These cells present antigen to T cells and may sense viral infection by pattern-recognition receptors, consequently providing coregulatory signals for virus-specific T cells. A role of these cell types in the immunobiology of HCV infection can be assumed from several experimental studies (reviewed in [8]). Unfortunately, to our knowledge, no epidemiological studies addressing genetic factors involved in macrophage or dendritic cell function have been published thus far.

NK cells are lymphoid cells with the ability to exert antiviral functions through secretion of antiviral cytokines or lysis of infected cells. The function of NK cells is regulated by several inhibitory and activating NK receptors. Inhibitory receptors of NK cells that interact with target cells are of particular importance to avoid NK cell cytotoxicity. Importantly, killer cell immunoglobulin-like receptors (KIR) expressed by NK cells interact with certain HLA class I molecules expressed by target cells (Table 1). Thus, it can be hypothesized that a genetic background that favors activating NK cell signals due to weaker inhibitory interactions of KIR receptors and HLA class I ligands or the expression of activating KIR-HLA pairs might support HCV clearance [9].

The majority of identified KIR ligands is composed of HLA-C alleles (Table 1). These alleles can be grouped depending on their binding characteristics into HLA-C1 and HLA-C2. Importantly, the strongest inhibitory signals of KIR-HLA pairs have been observed for HLA-C2 ligands. Presence of two HLA-C2 alleles is therefore likely to result in stronger inhibitory NK cell signals since this genomic background excludes interactions with HLA-C1 alleles. Interestingly, it has been shown that HLA-C2C2 alleles are enriched in patients with chronic HCV infection, while HLA-C1C1 alleles are associated with viral clearance [10, 11]. This suggests that genes regulating the activation of NK cells may have an impact on the outcome of HCV infection. In line with this, HLA-Cw*01 and HLA-Cw*3 (HLA-C1 group) were found to be associated with viral clearance while HLA-Cw*04 (HLA-C2 group) was associated with viral persistence [11–14]. However, recently, HLA-Cw*05 (HLA-C2 group) was found to be protective [15]. A critical role for NK cells in HCV infection is further supported by the finding that NK cells are inhibited by the HCV envelope glycoprotein E2, indicating that evasion of antiviral NK cell responses might be advantageous to the virus [16, 17]. However, this view is challenged by experimental evidence from an *in vitro* cell culture system, in which there was no inhibition of NK cells by HCV virions [18]. Therefore, the current understanding that high-dose infection including high levels of E2 might be able to inhibit NK cell function and result in viral persistence has to be reevaluated. Nevertheless, there is some evidence that clearance of low-dose infection may be related to NK cell responses. Indeed, expression of KIR2DL3 and ligand HLA-C1, that generate only weak inhibitory signals, were found to confer protection in a cohort with low-dose exposure to infection [10, 11]. Another study identified a decrease of KIR2DL2 (intermediate inhibitory signal when engaged by HLA-C1) and KIR2DS2 accompanied by an increase of the activating receptor KIR2DS5 (unknown ligand) in patients who cleared the virus [19]. In the same study, an increased frequency of the activating receptor KIR2DS3 (unknown ligand) was found to be associated with high levels of liver transaminases in patients with chronic HCV infection. In addition, the presence of two copies of the activating receptor-ligand pair KIR3DS1 and HLA-Bw4 was markedly enriched in patients with chronic HCV that had progressed to cirrhosis. Interestingly, these alleles were protective against HCC development in a Spanish cohort of patients [20]. These studies indicate that combinations of KIR and HLA class I molecules may result in a low activation of NK cells and may thus be beneficial in the initial phase of low-dose-infection. However, if the innate immune response is overwhelmed (as it might generally be in high-dose infection), activating KIR-HLA interactions may result in increased NK cell activity with increased liver damage and progression to liver fibrosis or cirrhosis.

Next to receptor-ligand interactions, the function of NK cells can be inhibited by transforming growth factor (TGF)-β [21]. Interestingly, polymorphisms in the promoter region of the TGF-β1 gene that result in a reduced expression of TGF-β1 have been associated with HCV clearance [22, 23]. These findings support the notion that NK cells that are not inhibited by TGF-β1 may be protective in HCV infection. This is in line with the finding that polymorphisms associated with higher levels of TGF-β1 production are associated with viral persistence [24, 25]. In addition, TGF-β1 gene polymorphisms have been found to influence the viral load in chronic HCV infection [26]. However, TGF-β1 is likely to have several other, non-NK cell related effects, for example a pivotal role in fibrogenesis [27]. Indeed, polymorphisms associated with high production of TGF-β1 are a risk factor for progressive hepatic fibrosis [24].

In sum, genes involved in innate immune responses are associated with different outcomes of HCV infection. Particularly genes involved in balancing the NK cell activation threshold may play an important role in HCV natural history.

### 2.2. Genes involved in adaptive immunity

#### 2.2.1. HLA class I

CD8+ T cell responses play a pivotal role in the outcome in HCV infection. Viral clearance is associated temporally with the presence of polyfunctional, multispecific CD8+ T cell responses, while the absence or impairment of CD8+ T cell responses results in viral persistence. CD8+ T cell responses depend on the interaction of virus-specific TCRs with viral peptides bound to HLA class I molecules presented by antigen-presenting cells (e.g., virus-infected hepatocytes). The HLA *locus* displays a high genetic variability in humans. One important distinguishing feature between the multitude of HLA alleles are polymorphisms in the peptide binding region that determine which peptides can be bound and presented to T cells. Thus, different HLA alleles are able to bind and present different viral epitopes, likely influencing both the quantity and the quality of the antiviral T cell response. While a high number of potential epitopes presented by an HLA allele may be advantageous, viral escape mutations may occur that evade the immune response in several epitopes and abrogate this protective effect. However, since viral mutations confer a varying degree of replicative fitness cost to the virus, HLA alleles able to present viral epitopes that cannot mutate easily are likely to be beneficial to the host.

Several associations between HLA class I alleles and the natural course of HCV infection have been described in a large number of studies worldwide (Table 2). These studies varied significantly in design (e.g., patients with chronic HCV *vs.* controls, viral clearance *vs.* controls, outcome after single-source infection) and cohort characteristics (e.g., ethnic background, route of infection, gender, age) which may account for some contradictory findings. However, there is general agreement that certain HLA class I alleles are associated with protection. For example, in a large study by Thio *et al.* in Caucasians and black Americans [13], HLA-A1101 and HLA-B57 were found significantly more often in 231 individuals with well-documented HCV clearance compared to 444 matched chronically infected patients. HLA-B57 was also found to be protective in a West African population [28]. Some HCV-specific CD8+ T cell epitopes have been identified that are restricted by HLA-B57 [29, 30], however, the mechanisms that contribute to protection have not been identified thus far. In an analysis of Irish women who had been inoculated from a single source during rhesus prophylaxis, McKiernan *et al.* identified HLA-A03 and HLA-B27 as alleles protecting from chronic HCV infection [12], of which HLA-B27 showed the strongest association with protection (OR = 7.99). Interestingly, the protective role of HLA-B27 could be linked to a CD8+ T cell epitope that was targeted by the majority of individuals with resolution of infection [31]. Importantly, viral escape mutations in the HLA-B27 binding anchors of this epitope result in a profound viral fitness cost and thus do not occur [32]. Hence, viral escape mutations occur in non-anchor binding sites, but a cluster of mutations is required to escape T cell recognition. These data reveal a protective role for HLA-B27 in HCV infection due to the generation of CD8+ T cell responses against a single epitope in which escape mutations are difficult for the virus to achieve. However, if viral escape mutations are preexistent (e.g., due to sequence variations in other viral genotypes or selection pressure on circulating strains in the population), the protective effect of HLA-B27 may be lost. It is possible that any observed HLA association may be due to linkage disequilibrium with other genes in proximity to the HLA *locus* that may influence the outcome of HCV infection. In the case of HLA-B27, however, the observation that the association with protection can be linked to a specific CD8+ T cell epitope derived from HCV genotype 1 but not genotype 3 (Neumann-Haefelin *et al.*, in revision) argues against a confounding role of linkage disequilibrium. The data regarding associations of other HLA class I alleles and HCV infection is less clear: HLA-B35 was found to be a protective allele in a Tunisian study population [33], but was associated with viral persistence in a Korean study [34]. A potential protective role for HLA-B8 was seen in a Saudi patient cohort [35] and HLA-B8 was underrepresented in a large cohort of patients with chronic HCV infection who received a liver transplant [36], however, HLA-B8 was clearly associated with viral persistence in the Irish study population [12]. This discrepancy may be due to preexistent escape mutations in the immunodominant HLA-B8 restricted T cell response in the Irish cohort [37]. In addition, several other HLA class I alleles have been associated with viral persistence (Table 2).

In sum, several HLA class I alleles are associated with different outcomes of HCV infection. Clearly at this point, associations between HLA alleles and outcome are still primarily descriptive. The determination of mechanisms behind theses associations is crucial to understand potential causal relationships of the observed HLA associations. Among the protective alleles, HLA-B27 plays a prominent role since the mechanisms of protection can be tracked down to the generation of HCV-specific CD8+ T cell responses against a single viral epitope [31]. This clearly demonstrates the influence of the genetic HLA class I background on the natural course of HCV infection.

#### 2.2.2. HLA class II

CD4+ T cells play an important role in the immune response against viral infections. Functions of CD4+ T cells include, among others, the provision of supportive signals to CD8+ T cells and B cells, polarization of the immune response and. to a minor degree, direct antiviral efficacy [41]. Lack of CD4+ T cells results in the inability to control HCV infection in an animal model of HCV [42]. During acute HCV infection in humans, only weak and monospecific CD4+ T cell responses are detectable in patients with evolving viral persistence while strong and multispecific CD4+ T cell responses can be detected in acute resolving disease [1]. CD4+ T cells are activated through the binding of the TCR to peptides presented by HLA class II molecules (HLA-DP, -DQ and -DR in humans). The peptide-binding region of the HLA class II molecules is formed by both the alpha and the beta chain. Thus, both gene *loci* may contribute to differences in peptide binding and one individual may have four different types of HLA-DQ and HLA-DR molecules. Comparable to HLA class I molecules, different HLA class II molecules bind to different viral peptides which may modulate the antiviral T cell response depending on the genetic background of the individual.

Several associations between HLA class II alleles and the outcome of HCV infection have been identified (Table 3). DQB1*0301 was associated with clearance of HCV infection in studies conducted in populations throughout the world [43–49]. Interestingly, DQB1*0301 is in close linkage disequilibrium (non-random association between polymorphisms at different *loci*) with DRB1*1101 and, indeed, DRB1*1101 is also associated with HCV clearance in several studies [13, 14, 43–45, 49–52]. Given the data, it may seem surprising that DRB1*1101 was not identified as a protective HLA allele in the well-documented Irish cohort that originated from a single source infection [53]. In addition, only a trend towards a protective role of DQB1*0301 was seen in that cohort [53]. Instead, DRB1*01 and DQB1*0501 were associated with viral clearance in several studies performed in the Irish cohort [12, 53–56]. Interestingly, the protective role of DRB1*01 and DQB1*0501 was also found in studies performed in the USA, but only among whites [13, 15]. A study in Puerto Rico found an association of DQB1*0501 and viral persistence [11]. It is intriguing to speculate that the protective effects of DRB1*01 and DQB1*0501 might be restricted to a population of Irish descent, including people that migrated from Ireland to the United States in the past centuries. In the Irish cohort, DRB1*0401 and DRB1*15 were identified as additional alleles associated with viral clearance [12]. For DRB1*15, the same effect was found in patients from central Europe [47, 57, 58]. Different roles for the contribution of HLA class II alleles to the natural history of HCV infection may exist between European and Asian populations. While DRB1*0701 was associated with viral persistence in Irish, Polish and mixed European populations, the same allele was associated with viral clearance in a Thai cohort [47, 56, 58, 59]. In addition, contrary associations of DRB1*0301 have been described in European and Asian cohorts [34, 47, 59, 60]. These results indicate that allelic associations with the outcome of HCV infection may vary depending on the ethnic background of the study population. Little is known about the mechanisms that determine the role of HLA class II alleles in HCV infection. Immunodominant HCV-specific CD4+ T cell epitopes (epitopes targeted by the majority of patients) were identified in patients with acute HCV infection [61, 62]. Interestingly, the sequence of these epitopes was highly conserved among the genotypes analyzed. Notably, the immunodominant epitopes were restricted by HLA class II alleles with known protective associations (DRB1*04, DRB1*11, DRB1*15 and DQB1*0301). Furthermore, *in vitro* binding studies revealed that most peptides also bound with high affinity to the DRB1*0101 allele, which was associated with protection in the Irish cohort. Nevertheless, these CD4+ T cell responses were infrequently detected in other studies [63, 64]. In a recent study that analyzed the magnitude of CD4+ T cell responses in an anti-HCV positive cohort, no correlation between the outcome of HCV infection and the magnitude of CD4+ T cell responses was found, despite a clear association of the outcome of HCV infection with the alleles DRB1*11, DQB1*03 and DRB3*02 [49]. This indicates that the CD4+ T cell responses detected do not fully explain the associations of HLA class II alleles and the outcome of HCV infection. However, it should be noted that CD4+ T cells are heterogenous, including regulatory T cells, TH1, TH2 and TH17 cells [41]. Since detection of CD4+ T cell responses in the above studies was performed largely by functional tests skewed to detect TH1 responses, it is possible that the contribution of other CD4+ T cell subsets could not be assessed. Indeed, regulatory T cells are enriched in chronic HCV infection compared to healthy controls and after viral clearance [65–69]. Furthermore, TH17 cells may play a role in the immunobiology of HCV infection [70]. It is thus possible that certain HLA class II alleles may modify the repertoire of these T cell populations and contribute to the outcome of HCV infection. Clearly, additional studies will be needed to address the immunological mechanisms that determine the protective or detrimental effects of HLA class II alleles in HCV infection.

Taken together, several HLA class II alleles have been associated with viral clearance or persistence in HCV infection. However, these results are quite variable in study populations of different ethnic backgrounds and the reasons for these findings have not been identified so far. In addition, the mechanisms determining the role of HLA class II alleles in HCV infection have yet to be elucidated.

### 2.3. Genes involved in T cell regulation and function

Suppression of effector T cell functions may be beneficial for the host since it limits overwhelming immunopathology. Of note, this may explain the high frequency of regulatory T cells observed in chronic HCV infection (reviewed in [74]). Regulatory T cells have the ability to suppress cytotoxic T cell responses by cell-cell contact and the production of immunosuppressive cytokines (e.g., IL-10). Next to regulatory T cells, other non-T cells can produce IL-10 (e.g., monocytes, dendritic cells). The suppressive function of IL-10 on T cell responses during viral infections has been demonstrated in mice [75, 76]. Viral persistence and high levels of viremia were associated with high levels of IL-10 and exhaustion of virus-specific CD8+ T cells. Importantly, blockade of IL-10 resulted in viral clearance and reversal of the T cell exhaustion. Interestingly, IL-10 was also identified as a soluble factor involved in the suppression of T cell responses in the livers of patients with HCV infection [77]. Several studies have analyzed the role of promoter polymorphisms that result in an altered production of IL-10 in HCV infection. Indeed, viral clearance was associated with polymorphisms that result in a low production of IL-10 [78–80]. Similarly, low levels of IL-10 production by monocytes were associated with viral clearance [81]. In contrast, polymorphisms associated with high IL10 levels were associated with viral persistence [78–80]. This is in agreement with the finding that higher IL-10 levels were detected in chronic HCV infection compared to controls [82]. This finding may possibly be explained by experimental evidence *in vitro* that HCV induces IL-10 production [83]. However, an association of IL-10 polymorphisms with the outcome of HCV infection was not seen in other studies [23, 82] or only in certain genetic ethnic groups (e.g., black Americans, but not Caucasians) [84]. These results support the hypothesis that the reduced inhibition of antiviral T cell responses by low IL-10 levels may result in enhanced viral clearance, while in contrast, high IL-10 levels are associated with viral persistence. A role for IL-10 in HCV immunobiology is further supported by a study that analyzed IL-10 receptor polymorphisms and found associations with different outcomes of HCV infection [85]. In sum, gene polymorphisms associated with IL-10 production and signaling most likely affect the outcome of HCV infection due to altered immunoregulatory functionality.

The function of virus-specific CD8+ T cells is an important parameter that determines the outcome of HCV infection. In a recent study, polyfunctional HCV-specific T cells that were able to produce antiviral cytokines, to secrete cytotoxic granula and possessed higher levels of anti-apoptotic molecules were associated with viral clearance, while T cells with few antiviral functions were associated with viral persistence [86]. Only few HCV-specific CD8+ T cell functions are seen in chronic infection in the liver, which indicates that impairment of CD8+ T cell functions may be an important determinant for viral persistence [87]. Polymorphisms influencing the expression of antiviral cytokines have been analyzed in several studies [23, 82, 88–92]. For example, an association between a polymorphism in the TNF gene with the outcome of HCV infection was found in a Taiwanese cohort [82], but not in other studies [23, 25, 80, 92]. No influence of IFN-γ gene polymorphisms on HCV natural history was noted in several studies [23, 25, 80], however, a single nucleotide polymorphism in the proximal IFN-γ promoter region that conferred higher promoter activity was associated with spontaneous recovery from HCV infection [93]. Hence, the role of genetic factors in the impairment of T cell responses in chronic HCV infection is not clear to date.

The function of CD8+ T cells depends on the maturation stage, which can be assessed by the combination of several differentiation markers linked to T cell functions [94]. Naïve T cells express a large isoform of the protein tyrosine phosphatase CD45, termed CD45RA. Upon activation, expression of this isoform is down-regulated in T cells and a short isoform, CD45RO, is expressed. Reexpression of CD45RA may occur in antigen-experienced CD8+ T cells, but is associated with late differentiation stages that have impaired proliferative capacity [94]. Interestingly, the CD45 gene polymorphism C77G was more frequent in patients with HCV infection compared to the overall population [95]. This point mutation results in the coexistence of both CD45RA and CD45RO splicing variant expression and may influence T cell signaling [95]. Alterations in the differentiation of virus-specific CD8+ T cells have been identified in patients with chronic HCV infection [29, 96–99]. However, to date no study has addressed the influence of the C77G polymorphism on HCV-specific CD8+ T cells. The differentiation of CD4+ T cells is influenced by several cytokines that promote polarization of CD4+ T cell responses (e.g., into TH1 or TH2) [41]. IL-12 is a cytokine that is prominently involved in the polarization of CD4+ T cells into TH1 cells [100]. Interestingly, a protective association of polymorphisms in the IL-12B gene has been identified in a cohort that was exposed to HCV but was not infected [101]. Also, polymorphisms in the promoter of the proinflammatory cytokine IL-18 were linked to viral clearance in a cohort of African American drug users [102]. These studies indicate that differences in the polarization of the T cell response due to the individuals’ genetic background may have an impact on the natural course of HCV infection.

Chemokine receptors play an important role in T cell differentiation and function, regulating migration and T cell effector functions. Chemokines are likely to play an important role in HCV infection, but only few genetic studies addressing the chemokine/chemokine receptor system have been performed to date [103]. A CCR2 polymorphism was associated with viral clearance in one report [104], but could not be confirmed by another [105]. Interestingly, a study in a German cohort found a higher prevalence of homozygosity of the HIV protective CCR5delta32 polymorphism in patients with chronic HCV infection [106]. However, heterozygosity of this polymorphism was protective in the well-defined Irish cohort [107]. CCR5delta32 was associated with reduced liver inflammation [107–110], indicating a role of genetic alterations of CCR5 in the outcome and progression of HCV infection. However, no association of different CCR5 gene alleles with viral persistence was found in several other cohorts [104, 109, 111, 112]. A recent study addressed the effects of CCR5delta32 mutation on HCV specific T cell responses [113]. IFN-γ responses were reduced in patients carrying the mutation but other T cell functions (migration, proliferation, IL-4 production) were not altered. Taken together, it is unclear whether CCR5delta32 mutations play a significant role in HCV infection. Clearly, it is less important than in HIV infection, where CCR5delta32 confers resistance to infection.

In sum, genes associated with T cell regulation and function have been reported to influence the outcome of HCV infection. Specifically, polymorphisms involved in the suppression of T cell responses by IL-10 may affect the natural history of HCV infection. Further studies will be needed to clarify the relevance of genetic alterations for other molecules important for T cell functions and/or differentiation.

## 3. Conclusions

A large number of studies have analyzed the influence of the host genetic background on the natural history of HCV infection. The strongest impact was found for factors involved in the immune response, particularly the CD8+ T cell response. In the interaction between virus and host, these protective alleles may determine the success of the overall antiviral immune response. It is important to note that viral factors are also likely to play a role in determining the impact of the host genetic background on the natural history of HCV infection. For example, different HCV genotypes carry different peptide sequences within T cell epitopes and HLA alleles with protective effects associated with one genotype may not be advantageous when challenged by another genotype. Nevertheless, the evidence obtained thus far indicates that the genetic background of the innate and adaptive immune response may significantly affect the natural history of HCV infection. A better understanding of the role of the host genetic background in patients with HCV infection is crucial for the development of new prophylactic and immunomodulatory antiviral strategies.

## Figures and Tables

**Table 1.** KIR receptors and their ligands (modified from [9]).

**KIR gene**	**Signalling**	**Ligand**
2DL1	Inhibiting	HLA-C2
2DL2	Inhibiting	HLA-C1
2DL3	Inhibiting	HLA-C1
2DL4	Activating	HLA-G
2DL5	Inhibiting	not known
3DL1	Inhibiting	HLA-Bw4
3DL2	Inhibiting	HLA-A3, HLA-A11
2DS1	Activating	HLA-C2
2DS2	Activating	HLA-C1
2DS3	Activating	not known
2DS4	Activating	not known
2DS5	Activating	not known
3DS1	Activating	HLA-Bw4
3DL3	not known	not known

**Table 2.** Associations between HLA class I alleles and HCV outcome.

**HLA-Type**	**Cohort**	**Association / Virological Effect**	**Reference**
HLA-A03	Irish	Viral Clearance	McKiernan 2004 [12]
HLA-A03	American	Viral Clearance in Blacks	Wang 2009 [15]
HLA-A03	Korean	Persistence	Yoon 2005 [34]
HLA-A1101	American	Viral Clearance	Thio 2002 [13]
HLA-A19	Saudi	Persistence	Hadhoud 2003 [35]
HLA-A2	American	Clearance in Blacks, Persistence in Whites	Wang 2009 [15]
HLA-A2301	American	Persistence	Thio 2002 [13]
HLA-A28	Egyptian	Persistence	Zekri 2005 [38]
HLA-A29	Egyptian	Persistence	Zekri 2005 [38]
HLA-B14	Egyptian	Persistence	Zekri 2005 [38]
HLA-B14	Italian	Persistence	Zavaglia 1996 [39]
HLA-B27	Irish	Viral Clearance	McKiernan 2004 [12]
HLA-B35	Tunisian	Viral Clearance	Ksiaa 2007 [33]
HLA-B35	Korean	Persistence	Yoon 2005 [34]
HLA-B46	Korean	Persistence	Yoon 2005 [34]
HLA-B57	American	Viral Clearance	Thio 2002 [13]
HLA-B57	African	Viral Clearance	Chuang 2007 [28]
HLA-B61	Japanese	Persistence	Higashi 1996 [14]
HLA-B8	Saudi	Viral Clearance	Hadhoud 2003 [35]
HLA-B8	Irish	Persistence	McKiernan 2004 [12]
HLA-C1C1	USA+UK	Viral Clearance	Khakoo 2004 [10]
HLA-C1C1	Puerto Rican	Viral Clearance	Romero 2008 [11]
HLA-C2C2	USA+UK	Persistence	Khakoo 2004 [10]
HLA-Cw*05	American	Viral Clearance	Wang 2009 [15]
HLA-Cw*3	Japanese	Persistence	Higashi 1996 [14]
HLA-Cw01	Irish	Viral Clearance	McKiernan 2004 [12]
HLA-Cw0102	American	Viral Clearance	Thio 2002 [13]
HLA-Cw04	American	Persistence	Thio 2002 [13]
HLA-Cw04	Irish	Persistence	Fanning 2004 [40]

**Table 3.** Associations between HLA class II alleles and HCV outcome.

**HLA-Type**	**Cohort**	**Association / Virological Effect**	**Reference**
DQA1*0103	German	Viral Clearance	Hohler 1997 [60]
DQA1*0201	Thai	Viral Clearance	Vejbaesya 2000 [59]
DQA1*03	Caucasian	Viral Clearance	Cramp 1998 [50]
DQA1*03	Northern European	Viral Clearance	Tibbs 1996 [71]
DQA1*03	Caucasian/UK	Viral Clearance	Cramp 1998 [50]
DQA1*0501	Korean	Viral Clearance	Yoon 2005 [34]
DQB1*02	French	Persistence	Alric 2000 [43]
DQB1*02	American	Persistence	Wang 2009 [15]
DQB1*0201	Irish	Persistence	McKiernan 2000 [53]
DQB1*0201	Thai	Persistence	Vejbaesya 2000 [59]
DQB1*0201	Korean	Viral Clearance	Yoon 2005 [34]
DQB1*03	American	Viral Clearance	Wang 2009 [15]
DQB1*03	American	Viral Clearance	Harris 2008 [49]
DQB1*0301	French	Viral Clearance	Alric 1997 [44]
DQB1*0301	Caucasian	Viral Clearance	Cramp 1998 [50]
DQB1*0301	Caucasian	Viral Clearance	Minton 1998 [45]
DQB1*0301	Italian	Viral Clearance	Mangia 1999 [51]
DQB1*0301	Europeans	Viral Clearance	Thursz 1999 [47]
DQB1*0301	French	Viral Clearance	Alric 2000 [43]
DQB1*0301	American	Viral Clearance	Thio 2002 [13]
DQB1*0301	Italian	Viral Clearance	Zavaglia 1998 [72]
DQB1*0301	Polish	Viral Clearance	Wawrzynowicz 2000 [58]
DQB1*0301	Japanese	Viral Clearance	Higashi 1996 [14]
DQB1*0301	Caucasian/UK	Viral Clearance	Cramp 1998 [50]
DQB1*0302	Northern European	Viral Clearance	Tibbs 1996 [71]
DQB1*0303	Japanese	Viral Clearance	Higashi 1996 [14]
DQB1*05	American	Viral Clearance	Wang 2009 [15]
DQB1*0501	Irish	Viral Clearance	McKiernan 2000 [53]
DQB1*0501	American	Viral Clearance	Thio 2002 [13]
DQB1*0501	Puerto Rican	Persistence	Romero 2008 [11]
DQB1*0502	Italian	Viral Clearance	Congia 1996 [73]
DQB1*0601	Korean	Persistence	Yoon 2005 [34]
DQB1*0604	Korean	Persistence	Yoon 2005 [34]
DRB1*01	Irish	Viral Clearance	Barrett 1999 [55]
DRB1*01	Irish	Viral Clearance	Fanning 2000 [56]
DRB1*01	Irish	Viral Clearance	Barrett 2001 [54]
DRB1*01	American	Viral Clearance	Wang 2009 [15]
DRB1*0101	Irish	Viral Clearance	McKiernan 2000 [53]
DRB1*0101	American	Viral Clearance	Thio 2002 [13]
DRB1*0101	Irish	Viral Clearance	McKiernan 2004 [12]
DRB1*0301	Europeans	Viral Clearance	Thursz 1999 [47]
DRB1*0301	Thai	Persistence	Vejbaesya 2000 [59]
DRB1*0301	Korean	Viral Clearance	Yoon 2005 [34]
DRB1*0301	German	Persistence	Hohler 1997 [60]
DRB1*03011	Irish	Persistence	McKiernan 2000 [53]
DRB1*04	Caucasian/UK	Viral Clearance	Cramp 1998 [50]
DRB1*04	Caucasian/UK	Viral Clearance	Cramp 1998 [50]
DRB1*0401	Irish	Viral Clearance	McKiernan 2004 [12]
DRB1*0701	Europeans	Persistence	Thursz 1999 [47]
DRB1*0701	Irish	Persistence	Fanning 2000 [56]
DRB1*0701	Polish	Persistence	Wawrzynowicz 2000 [58]
DRB1*0701	Thai	Viral Clearance	Vejbaesya 2000 [59]
DRB1*08	Tunisian	Viral Clearance	Ksiaa 2007 [33]
DRB1*0803	Korean	Persistence	Yoon 2005 [34]
DRB1*11	Caucasian	Viral Clearance	Minton 1998 [45]
DRB1*11	American	Viral Clearance	Harris 2008 [49]
DRB1*1101	French	Viral Clearance	Alric 1997 [44]
DRB1*1101	Europeans/UK	Viral Clearance	Thursz 1999 [47]
DRB1*1101	French	Viral Clearance	Alric 2000 [43]
DRB1*1101	Italian	Viral Clearance	Scotto 2003 [46]
DRB1*1101	Turkish	Viral Clearance	Yenigun 2002 [48]
DRB1*1104	Italian	Viral Clearance	Mangia 1999 [51]
DRB1*1104	Italian	Viral Clearance	Zavaglia 1998 [72]
DRB1*12	American	Clearance in Blacks, Persistence in Whites	Wang 2009 [15]
DRB1*1201	Europeans	Viral Clearance	Thursz 1999 [47]
DRB1*1301	German	Viral Clearance	Hohler 1997 [60]
DRB1*15	Irish	Viral Clearance	McKiernan 2004 [12]
DRB1*1501	Europeans	Persistence	Thursz 1999 [47]
DRB1*1501	Polish	Persistence	Wawrzynowicz 2000 [58]
DRB1*15011	German	Viral Clearance	Lechmann 1999 [57]
DRB1*1601	Italian	Viral Clearance	Congia 1996 [73]
DRB3*02	American	Viral Clearance	Harris 2008 [49]
DRB4*0101	Europeans	Persistence	Thursz 1999 [47]
